# Inhibitory Potential of Resveratrol in Cancer Metastasis: From Biology to Therapy

**DOI:** 10.3390/cancers15102758

**Published:** 2023-05-14

**Authors:** Baohong Song, Wei Wang, Xuemei Tang, Robby Miguel Wen-Jing Goh, Win Lwin Thuya, Paul Chi Lui Ho, Lu Chen, Lingzhi Wang

**Affiliations:** 1 State Key Laboratory of Southwestern Chinese Medicine Resources, School of Pharmacy, Chengdu University of Traditional Chinese Medicine, Chengdu 611137, China; 2020KS351@stu.cdutcm.edu.cn (B.S.); wangwei@stu.cdutcm.edu.cn (W.W.); XMTang@stu.cdutcm.edu.cn (X.T.); 2 Cancer Science Institute of Singapore, National University of Singapore, Singapore 117599, Singapore; r.goh@nhs.net (R.M.W.-J.G.); csithuya@nus.edu.sg (W.L.T.); 3 School of Pharmacy, Monash University Malaysia, Subang Jaya 47500, Malaysia; paul.ho@monash.edu; 4 Department of Pharmacology, Yong Loo Lin School of Medicine, National University of Singapore, Singapore 117600, Singapore; 5 National University Cancer Institute, National University of Singapore, Singapore 119074, Singapore; 6 NUS Center for Cancer Research (N2CR), Yong Loo Lin School of Medicine, National University of Singapore, Singapore 117599, Singapore

**Keywords:** resveratrol, tumor metastasis, metastasis animal model, cancer therapy

## Abstract

**Simple Summary:**

The majority of cancer-related deaths are attributed to metastatic tumors, making the inhibition of cancer metastasis a critical challenge in cancer treatment. Resveratrol, a compound first reported in 1997 to have anticancer properties, has shown potential in suppressing cancer metastasis in preclinical studies. Despite these positive results, clinical trials have yielded limited progress so far, highlighting the need to understand the cellular processes and signaling pathways involved in resveratrol’s anticancer activity against metastatic tumors. This review article summarizes the past five years’ research on resveratrol’s potential in cancer prevention or therapy and signaling pathways in inhibiting cancer metastasis and evaluates its bioavailability and toxicity. We also discuss the challenges of using resveratrol as an anticancer drug candidate and the current animal models used for preclinical studies. Overall, this article provides valuable insights into the development of resveratrol as an antimetastatic drug for cancer therapy.

**Abstract:**

Cancer metastasis is a significant challenge in cancer treatment, and most existing drugs are designed to inhibit tumor growth but are often ineffective in treating metastatic cancer, which is the leading cause of cancer-related deaths. Resveratrol, a polyphenol found in grapes, berries, and peanuts, has shown potential in preclinical studies as an anticancer agent to suppress metastasis. However, despite positive results in preclinical studies, little progress has been made in clinical trials. To develop resveratrol as an effective anticancer agent, it is crucial to understand its cellular processes and signaling pathways in tumor metastasis. This review article evaluates the current state and future development strategies of resveratrol to enhance its potency against cancer metastasis within its therapeutic dose. In addition, we critically evaluate the animal models used in preclinical studies for cancer metastasis and discuss novel techniques to accelerate the translation of resveratrol from bench to bedside. The appropriate selection of animal models is vital in determining whether resveratrol can be further developed as an antimetastatic drug in cancer therapy.

## 1. Introduction

According to data from the American Cancer Society, approximately 1,958,310 new cases of cancer and 609,820 cancer-related deaths are projected to occur in the United States in 2023 [1]. Unfortunately, at the time of diagnosis, around 50% of cancer patients have already developed some form of metastasis, with metastatic disease being mostly incurable. It is reported that 90% of cancer-related deaths are caused by metastatic tumors, and the five-year survival rate for patients with metastatic disease is significantly lower than those with early stage cancer [2]. Cancer metastasis is a common characteristic of advanced cancer and a primary cause of treatment failure. Despite the focus on the inhibition of tumor growth in drug development, there has been limited progress in discovering drugs that target cancer metastasis. Natural compounds have been shown to be a valuable source for the development of novel anticancer drugs, as demonstrated by paclitaxel, which has been a first-line drug for the treatment of breast cancer since its FDA approval in 1992. Investigation of new phytochemicals from nature is therefore an important solution to finding effective anticancer treatments for cancer patients.

**Figure 1:**
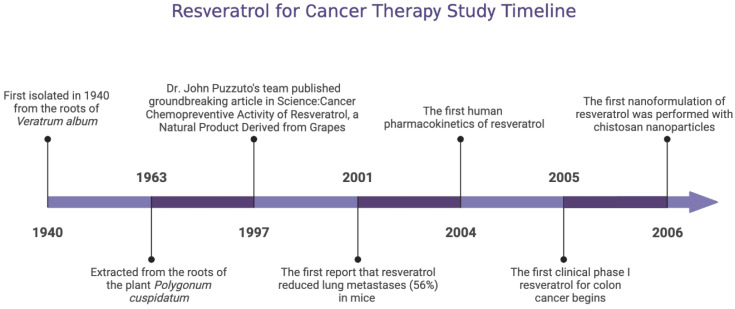
Timeline of resveratrol milestone events.

Resveratrol (3,4′,5-trihydroxy-trans-stilbene, RES) is a “miracle” nutraceutical that has been used in cancer prevention for decades. It is found naturally in foods such as peanuts and pistachios, and to a lesser extent in cranberries and blueberries [3]. RES exists in two isomeric forms (*cis* and *trans*), but the *trans*-isomer has been shown to be more potent than the *cis*-isomer due to the lower steric hindrance of its side chains [4,5]. In addition, the antioxidant and anticancer effects of its isomers, adducts, derivatives, and conjugates have been intensively studied [6]. Therefore, the medicinal use of its trans-isomer has been the subject of research. It was first discovered in the root of *Veratrum album* (Figure 1) and was later extracted in 1963 from the root of *Knotweed cuspidatum*, a plant used for centuries in traditional Asian medicine to treat inflammation and other ailments [7]. RES really came to the fore in 1997 when Dr. John Puzzuto’s team published a blockbuster scientific study in the journal Science, confirming the cancer chemopreventive activity of RES extracted from grapes [8].

In 2001, Kimura et al. were the first to report that RES administered at doses of 2.5 and 10 mg/kg resulted in a significant reduction (56%) in lung metastases in mice afflicted with highly metastatic Lewis lung cancer (LLC) [9]. Before and after this, many studies on RES emerged, including the preliminary exploration of RES pharmacokinetics. In 2004, the first comprehensive report on the pharmacokinetics of RES in humans was published by Dr. Walle et al. It has been shown that at least 70% of the RES is absorbed after oral administration based on the measurement of total radioactivity (RES plus its various metabolites in vivo) [10,11]. In 2005, the first clinical study with colon cancer patients was launched. Formulations of RES are processed to alter their bioavailability, and the first nanoformulation was reported in 2006 [12]. Subsequently, the upsurge of RES anticancer-related research continues to increase, but no breakthrough has been made so far.

In recent years, many research groups have published RES review articles focusing on cancer prevention and treatment. In this review, we attempted to highlight the role of RES in suppressing cancer metastasis in preclinical studies, the current scenario of clinical trials, and the bottleneck of RES to be developed as a potential antimetastatic drug for the treatment of advanced cancer. More importantly, we analyze animal models currently available to study drug candidates that inhibit cancer metastasis and suggest new strategies to accelerate drug development to improve treatment outcomes in metastatic cancer.

## 2. Literature Search

**Figure 2:**
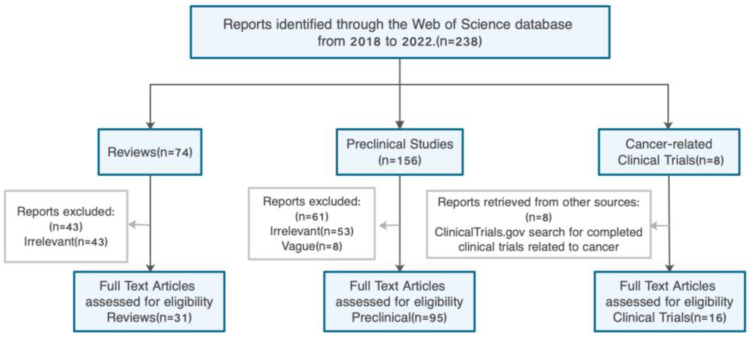
The primary literature search strategies for preclinical studies of resveratrol in cancer metastasis and clinical trials.

The literature was retrieved from the Web of Science database. The search time range is the past five years. The first search term was “resveratrol (Topic) AND cancer metastasis (Topic)”. To make the search results more comprehensive, a second search was performed using the term “resveratrol (Topic) AND tumor metastasis (Topic)”, as shown in Figure 2.

Clinical trials of resveratrol mentioned in the article were searched from ClinicalTrial.gov (https://clinicaltrials.gov, accessed on 1 June 2022). The search terms are “resveratrol (drug name) and cancer (disease)”.

A filter was applied for duplicates and the literature was summarized with EndNote X9. Subsequently, a screening process was conducted on the titles and abstracts, and any studies failing to meet the predetermined inclusion criteria were excluded. In the end, the entire body of literature was categorized into three distinct groups: reviews, preclinical studies, and clinical trials. These categories were subsequently subjected to another round of screening to identify the ones containing pertinent information and to evaluate their full texts for inclusion. Additional searches were conducted to retrieve additional information when necessary.

## 3. The Pathogenesis of Cancer Metastasis

**Figure 3:**
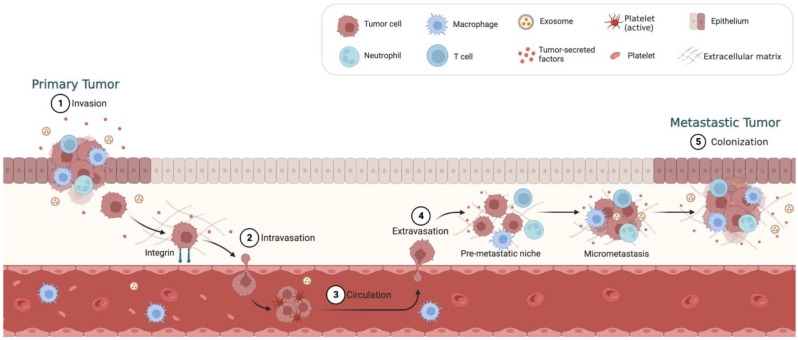
Summary of the pathologic process of cancer metastasis: The metastatic cascade is subdivided into multiple steps, including (1) invasion, (2) intravasation, (3) circulation, (4) extravasation, and (5) colonization.

Cancer metastasis is a complex process that involves multiple steps (Figure 3) [13]. The first step is the invasion of cancer cells into the surrounding tissue. Subsequently, the process of intravasation takes place, wherein cancer cells infiltrate and enter the bloodstream or lymphatic vessels. Following their exit from the primary site, cancer cells must evade immune surveillance and survive in circulation before they can establish a foothold in a distant organ through a process known as extravasation. Once extravasated, the cancer cells require a new blood supply to support their growth, which is facilitated by angiogenesis. Throughout the process of metastasis, cancer cells interact with the microenvironment of the distant site, including stromal cells such as fibroblasts, endothelial cells, and immune cells. These interactions can either promote or inhibit tumor growth and metastasis. Additionally, the extracellular matrix (ECM) plays a critical role in cancer metastasis. The ECM is a complex network of proteins and molecules that provide structural support for cells and regulate cell signaling. Cancer cells can modify the ECM to create a permissive environment for metastasis. The molecular mechanisms that underlie cancer metastasis are the subject of ongoing research. A number of genes and signaling pathways have been identified as playing critical roles in the process. For example, the epithelial–mesenchymal transition (EMT) is a process by which epithelial cells acquire mesenchymal properties, allowing them to become more motile and invasive. EMT has been linked to cancer metastasis and is regulated by several signaling pathways, including the Wnt, TGF-β, and Notch pathways. Other pathways implicated in cancer metastasis include the PI3K/Akt/mTOR pathway, the Rho GTPase pathway, and the NF-κB pathway.

## 4. Factors Associated with Cancer Metastasis

### 4.1. Angiogenesis

Angiogenesis is a physiological process that involves the development of new blood vessels from pre-existing ones. This intricate and multistep process entails the activation of endothelial cells, proliferation of cells, invasion, chemotactic migration, and differentiation into newly formed blood vessels [14]. Tumors require the development of new blood vessels to ensure an adequate supply of oxygen and nutrients for the tumor cells to grow and thrive. Furthermore, endothelial cells have the capability to produce growth factors through both autocrine and paracrine signaling pathways, which can effectively promote the proliferation of tumors [15]. The onset of angiogenesis coincides with an increase in the number of tumor cells entering the circulation, thereby promoting metastasis. Studies have consistently shown that for solid tumors, the transition from carcinoma in situ to invasive carcinoma must be accompanied by neovascularization [16,17,18]. Hence, the strategy of inhibiting angiogenesis could be an effective approach for treating cancer metastasis.

### 4.2. Epithelial–Mesenchymal Transition

Epithelial–mesenchymal transition (EMT) refers to a biological phenomenon in which epithelial cells undergo *trans*-differentiation and acquire the characteristics of mesenchymal cells. This process occurs under specific physiological and pathological conditions. During this process, epithelial cells lose cell polarity and cell adhesion, acquire migration and invasion characteristics, and undergo EMT, which is considered as the initial step of tumor metastasis [19,20]. EMT is characterized by a series of cellular events, including the breakdown of tight junctions, loss of apical-basal polarity, and rearrangement of the cytoskeletal structure. These changes enable tumor cells to acquire an aggressive phenotype. In the context of tumor development, the regulation of EMT is often dysregulated and influenced by various extracellular factors within the tumor microenvironment, including growth factors, inflammatory cytokines, and physical stressors such as hypoxia [21]. The process of EMT is intricately designed to facilitate the ability of tumor cells to acclimatize and adapt to the dynamic microenvironment of various tumors, ultimately promoting effective metastasis. One of the important features of the EMT process in solid tumors is the loss of function of E-cadherin expression, basic helix-loop-helix proteins (Twists), and forkhead box proteins (FOXCs) in tumor cells [22]. In addition, other transcripts involved in the EMT process include small non-coding RNAs, epigenetic regulators, and exogenous inducers. A variety of growth factors, the number of signaling pathways, and matrix metalloproteinases are also involved in the regulation of EMT [23]. In addition, cancer cells that undergo EMT show significant changes in morphology and molecular characteristics. These alterations include a reduction in the expression of epithelial markers, such as ZO-1 and occludin, as well as mesenchymal markers, such as N-cadherin, vimentin, and fibroblast-specific protein. These changes are further evidenced by the increased expression of fibronectin-1 [24].

### 4.3. Cancer Stem Cells

Tumor metastasis is not a capability shared by all cells within a tumor. Rather, cancer stem cells (CSCs) constitute a specific subset of cells that are capable of self-renewal, generating the diverse range of cells present within the tumor. CSCs play a role in tumor metastasis through direct or indirect involvement in the processes of angiogenesis and lymphangiogenesis, both of which are significant pathological changes associated with metastatic tumors [25]. Metastasis is driven by the evolution and selection of CSCs subsets and is considered the engine of cancer metastasis. CSCs were first identified in hematopoietic malignancies and later in a wide range of solid tumors, including breast, colon, and brain cancers [26]. Surface markers that facilitate cell interactions and provide them with unique properties have been used to identify CSCs [27]. Therefore, selectively targeting CSCs may be a promising therapeutic strategy against cancer metastasis.

### 4.4. Tumor Microenvironment

Tumor microenvironment (TME) is used to describe the specific cellular surroundings in which tumor or cancer stem cells are present [28]. In the 1990s, the “soil and seeds” theory of tumor metastasis proposed by Steven Paget was a milestone event in the history of TME research. He believed that tumor cells could colonize target organs only in a favorable microenvironment [29]. Subsequently, it was proposed that cancer cell seeds are intrinsically compatible with the microenvironmental soil of specific tissues, which helps determine metastatic organotropism [30]. The term “pre-metastatic niche” (PMN) was introduced in 2005 by Dr. Lyden and colleagues to describe the mechanism by which primary tumors attract bone marrow-derived cells to remote organs and create microenvironments that facilitate metastasis [31]. TME encompasses various components, including immune cells, blood vessels, extracellular matrix (ECM), fibroblasts, lymphocytes, myeloid-derived inflammatory cells, and signaling molecules. Through intricate interactions between the cellular and structural constituents of the TME, cancer cells gain the ability to invade surrounding tissue and propagate through a multistep metastatic process to distant sites. Thus, the TME plays a pivotal role in facilitating cancer cell metastasis. Tumor-associated macrophages (TAMs) are the main component of tumor leukocyte infiltration and play an important role in tumor metastasis [32]. The TME determines the interconversion between M1-type (“classically activated”) and M2-type (“alternately activated”) macrophages [33]. M1 macrophages elicit a response to cytokines, such as interferon-γ (IFN-γ), that impedes tumor progression by producing pro-inflammatory and immunostimulatory cytokines, including IL-12 and TNF-α. However, in various types of tumors, M2-type macrophages comprise the predominant TAMs. M2 macrophages possess immunosuppressive and growth-promoting properties, while mesenchymal cells produce copious exosomes to enhance the migratory capacity of cancer cells. Additionally, cancer-associated fibroblasts (CAFs) restructure the neighboring matrix and establish migration-promoting pathways for cancer cells [34].

### 4.5. Inflammation

Inflammation is a biological response that the body utilizes to counteract infection, tissue injury, or other forms of cellular stress and to promote tissue repair through restorative mechanisms [35]. A growing body of clinical and preclinical evidence accumulated over the past two decades has demonstrated that inflammation is a critical immune and reparative response that is indispensable for metastasis [36]. Inflammation is a hallmark of nearly all types of cancer and involves reciprocal communication between malignant and non-malignant cells through mediators such as cytokines, chemokines, and genetic changes. Ultimately, the inflammatory tumor microenvironment fosters tumor progression and metastasis. Interestingly, inflammation and EMT interact and maintain each other by forming a positive feedback loop. The most prominent inflammatory mediators that favor EMT and thus drive tumor cell migration, invasion, and metastatic potential include IL-1β, IL-6, IL-8, TNF-α, and some chemokines, such as CC chemokine ligand (CCL)2, CCL5 and CCL18 [37].

### 4.6. Genetic and Epigenetic Factors

Genetic mutations are thought to be one of the primary factors that facilitate metastatic events. Nevertheless, comprehensive sequencing studies have indicated that genetic mutations alone may not be sufficient to account for metastasis. Increasingly, it is recognized that epigenetic alterations play a crucial role in conferring additional characteristics on primary cancer cells that significantly contribute to the metastatic process [38]. Epigenetic modifications that promote metastatic progression occur by modifying the output of already activated transcriptional programs. The drivers of these programs are either specifically activated in cancer cells via oncogenic mutations or represent endogenous genetic and other factors that are not induced by cancer-specific oncogenic alterations. For example, hypermethylation at the loci of tumor suppressor genes, such as TP53, APC, and VHL, is frequently observed and associated with transcriptional silencing [39]. In addition, miRNAs can act as oncogenes with specific functions in angiogenesis, invasion, and migration, leading to cancer metastasis [40].

### 4.7. Extracellular Vesicles

Extracellular vesicles (EVs) are a type of vesicle that is released by all living cells and is composed of a bilayer lipid membrane. EVs are thought to be a means of conveying cellular information in large quantities during cancer metastasis [41]. EVs encompass multiple subtypes, including exosomes, microvesicles (MVs), endosomes, and apoptotic bodies [42]. In cancer biology, the role of EVs is now recognized as fundamental to the progression of all cancer processes, with EV content promoting tumorigenesis and metastasis. Accumulating evidence suggests that EVs, through a molecular characterization of their DNA, RNA, and protein content, can be used as disease biomarkers, enabling liquid biopsies to be used for cancer diagnosis, prognosis, and treatment selection [43].

Exosomes, as a particular subtype of EVs, have garnered significant attention and are among the most extensively studied classes. They are the smallest EVs, ranging in size from 30 to 150 nm, and are released from cells after the fusion of multivesicular bodies (MVBs) with the plasma membrane [44]. Compared to normal cells, cancer cells tend to produce a higher quantity of exosomes, which can influence both local and distant microenvironments. In primary TME, there is local signaling between tumor cells and surrounding cells, whereas remote signaling exists between tumor cells and metastatic sites. The latter signal promotes PMN formation and enhances the growth of disseminated tumor cells during metastasis to distant organs [45]. Exosomes can be considered as “spreaders” or “carriers” that promote tumor progression and metastasis.

## 5. In Vivo Models of Metastatic Cancer

**Figure 4:**
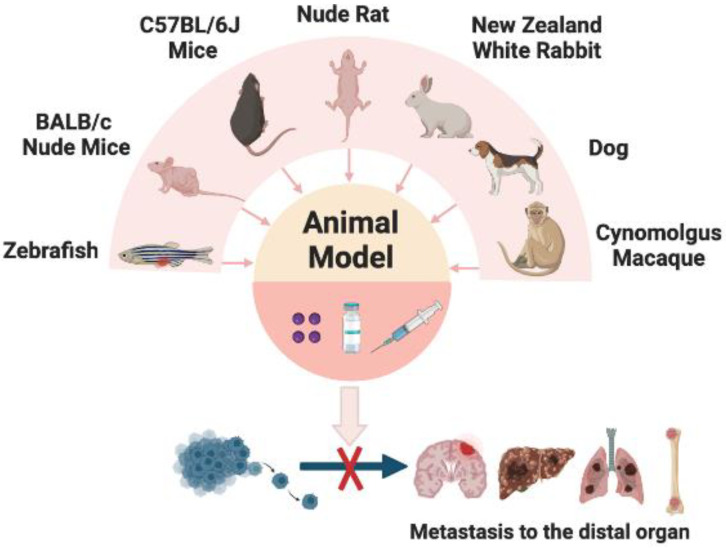
Animal models for the treatment of cancer metastasis and the site of administration.

The greatest challenge facing scientists is our incomplete understanding of the genetic and mechanical basis of complex human diseases, including cancer [46]. The use of well-established animal models has become and will continue to be an essential approach for studying the intricate interactions between cancer cells and the TME throughout the metastatic cascade [47]. Appropriate animal models should be used to address different aspects of cancer metastasis and to accelerate the development of antimetastatic drugs. Here, we summarize the animal models reported in research articles for the assessment of metastatic cancer (Figure 4).

The zebrafish (Danio rerio) has become a robust vertebrate model for investigating metastatic events in vivo. This is largely due to its unique attributes, including extrauterine development that facilitates embryonic manipulation and optically transparent tissues that enable real-time in vivo imaging of fluorescently labeled cells [48]. In addition, the zebrafish has been frequently used to study the molecular mechanisms of tumor metastasis due to its low-cost, high-throughput, and transparent and translucent characteristics. Chen et al. reported that a zebrafish xenograft model can be used to study human cancer stem cells and predict changes in the premetastatic tumor microenvironment to prevent cancer metastasis [49]. Although a zebrafish cancer metastasis model has not been reported in RES-related studies, Savio et al. reported the preventive effect of the RES analog 4,4′-dihydroxy-trans-stilbene (DHS) on cancer invasion and metastasis. DHS significantly inhibited the invasion of distant metastases of LLC cells in zebrafish embryos, with 0.1 μM DHS treatment significantly inhibiting metastasis (32%), but slightly lower than 1 μM DHS treatment (49%). However, 10 μM DHS was found to be toxic to zebrafish embryos [50].

Currently, rodents are the species most commonly used to assess metastatic cancer. The advantages of rodents are that they are close to humans in terms of body structure and function, rich in sources, cheap in price, moderate in size, and can be experimented with large samples, making the results more credible and convenient for research. Paschall et al. developed an orthotopic mouse model to investigate spontaneous metastasis in breast cancer [51]. A hair trimmer was used to shave the area surrounding the nipples. Following this, 4T1 tumor cells were injected into the mammary fat pad beneath the mammary glands of BALB/c mice. After waiting for a period of approximately 21–30 days and fully inflating the lungs with ink, small white dots representing tumor nodules were observed in the black lungs after a few minutes. The number of white dots was counted, with each dot representing a metastatic tumor nodule. After that, Jin et al. developed a novel method for assessing breast cancer metastasis by injecting eight barcoded lines as a pool into the left ventricle of SCID mice. Metastatic lesions were then detected using dual luminescence imaging (BLI) [52].

In colon cancer research, Morikawa et al. reported for the first time that human colon cancer cells could be injected into mice [53], thus successfully creating the first mouse intestinal cancer model and opening the exploration of colon cancer metastasis models. In the study conducted by Cespedes et al., nude mice were used to investigate human colorectal cancer cell lines (HCT-116, SW-620, and DLD-1). The cells were injected into the cecal wall of the mice at an angle of approximately 30°, with the tip inserted 5 mm. The bowel was then returned to the abdominal cavity and closed surgically after injection [54]. Liu et al. have adopted a novel approach for establishing an orthotopic animal model of lung cancer in BALB/c nude mice. Specifically, they have departed from the conventional methods of tail vein injection and intratracheal injection of cancer cells and opted for a direct inoculation of cancer cell suspension into the lungs, without the need for thoracotomy or intubation. Combined with dynamic spiral CT observation, it can detect the destination of cancer cell metastasis [55]. However, the authors also explained that the instrument is expensive and difficult to promote in scientific research. Furthermore, rats are ideal models for imaging brain metastases because their larger brains provide better relative spatial resolution compared to mouse brains. As summarized by Meulenaere et al., MRI was used to assess the formation of brain metastases in triple negative breast cancer (TNBC), while CT and 2-deoxy-2-[18F]fluoro-D-glucose ([18F]FDG) positron emission tomography (PET) imaging were enabled to assess bone metastases. However, nude mouse models are not recommended for studying brain metastases as a single disease, as mouse brains are too small to provide the spatial resolution when compared to that of rats [56].

The development of appropriate animal tumor models is a critical step in the preclinical evaluation of new drug candidates. These models are essential for simulating human disease conditions and assessing the safety and efficacy of potential therapeutic agents before proceeding to clinical trials. The use of mammalian models has enabled researchers to simulate and investigate various aspects of human cancer metastasis, providing valuable insights into the disease and informing the development of potential therapeutic interventions. Interestingly, dogs are the only species other than humans to spontaneously develop benign prostatic hyperplasia (BPH). The high incidence of cancer and subsequent osteoblastic bone metastasis makes it particularly valuable as a research model for the development of novel antimetastatic drugs [57]. Additionally, Mikael et al. concluded that canine osteosarcoma is generally considered a good clinically relevant model for human osteosarcoma, and in particular, dogs with idiopathic osteosarcoma are a clinically relevant model for studying early micrometastatic disease [58]. Knapp’s research suggests that canine bladder cancer is a valuable addition to experimentally induced rodent models for the study of bladder cancer. This is due to the similarities between naturally occurring bladder cancer in dogs and invasive bladder cancer in humans. In particular, high-grade invasive transitional cell carcinomas in dogs exhibit comparable biological behavior, metastatic patterns and frequency, as well as a similar response to therapy in terms of cellular and molecular characteristics [59]. Furthermore, rabbits have also been used as an animal model for studying metastases. Huang et al. conducted a study in which they injected VX2 squamous cell carcinoma into the left gastrocnemius muscle of 38 female New Zealand white rabbits via lumbar puncture and observed lung metastasis. The rabbits were divided into three groups and the lung metastases were evaluated on days 19, 22, and 25 after desquamation. The metastasis rates were found to be 33.3%, 38.5%, and 76.9%, respectively [60]. However, not every species can be developed as a transfer model. For example, Sun et al. attempted to establish an animal model of metastatic prostate cancer in rhesus monkeys that closely mimicked human disease characteristics, but were unsuccessful in doing so [61]. In conclusion, there are different in vivo models used to assess cancer metastasis. Each model has its own characteristics and can be chosen according to the purpose of the research project.

## 6. Resveratrol Inhibits Tumor Growth and Metastasis

### 6.1. In Vitro Studies of Resveratrol on Anticancer Activity

**Figure 5:**
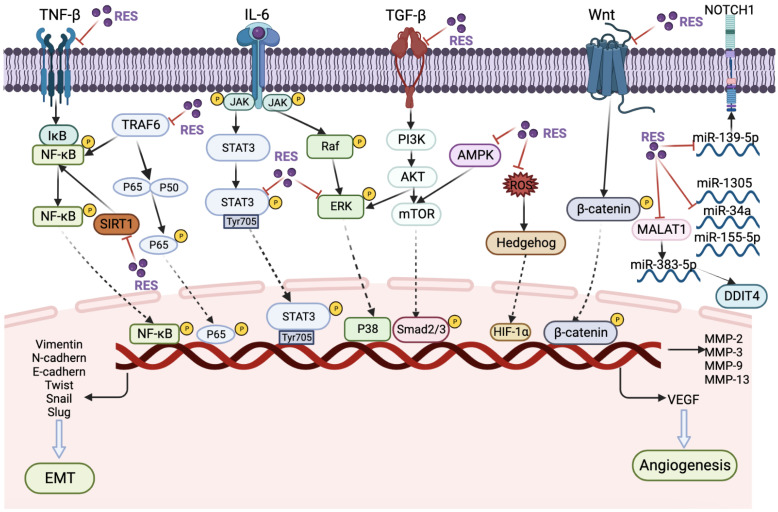
The schematic of the different signaling pathways of resveratrol in suppressing cancer metastasis. (***Abbreviations*:** AMPK, adenosine 5′-monophosphate-activated protein kinase; AKT, protein kinase B; DDIT4, DNA damage-inducible transcript 4; ERK, extracellular signal-regulated kinase; HIF-1α, hypoxia-inducible factor-1α; IL-6, interleukin-6; IκB, inhibitor of NF-κB; JAK, janus kinase; miR-1305, micro ribonucleic acid-1305; miR-139-5p, micro ribonucleic acid-139-5p; miR-155-5p, micro ribonucleic acid-155-5p; miR-383-5p, micro ribonucleic acid-383-5p; miR-34a, micro ribonucleic acid-34a; mTOR, mechanistic target of rapamycin; MALAT1, metastasis-associated lung adenocarcinoma transcript 1; MMP-2, matrix metalloproteinase-2; MMP-3, matrix metalloproteinase-3; MMP-9, matrix metalloproteinase-9; MMP-13, matrix metalloproteinase-13; NF-κB, nuclear factor-kappa B; NOTCH1, notch homolog 1; PI3K, phosphoinositide 3-kinases; ROS, reactive oxygen species; SIRT1, silent information regulator 1; STAT3, signal transducer and activator of transcription 3; TGF-β, transforming growth factor-β; TNF-β, tumor necrosis factor β;TRAF6, TNF-receptor associated factor 6; VEGF, vascular endothelial growth factor).

Following Dr. John’s seminal report in 1997 on the potential of RES to prevent cancer development and inhibit tumor growth, there has been a growing body of scientific literature investigating the use of RES as a chemopreventive agent or as a potential candidate for anticancer drug development. Despite these efforts, the mechanism by which RES inhibits cancer metastasis remains poorly understood and rarely reported. In this context, we have compiled a summary of preclinical studies on RES’s inhibitory effects on cancer metastasis, which is presented in Figure 5 and Table 1, along with the underlying signaling pathways.

Studies have demonstrated that RES can hinder cancer metastasis via the NF-κB pathway, a pivotal transcription factor that regulates the capacity of malignant and precancerous cells to evade programmed cell death-based tumor surveillance pathways [62]. In a recent in vitro model, Constance et al. reported that 3D alginate HCT116 cells in multicellular TME cultures consisting of fibroblasts and T lymphocytes can be used to investigate the impact of TNF-β, Sirt1-ASO and/or RES on colorectal cancer proliferation and invasion with a focus on cancer stem cells. The study revealed that RES inhibited the Sirt1 axis through the regulation of paracrine agent secretion and NF-κB signaling pathway. Additionally, it decreased the production of T lymphocyte/fibroblast (TNF-β, TGF-β3) proteins, thereby highlighting the potential of RES in preventing colorectal cancer metastasis [63]. In addition, another study demonstrated that treatment with 5 μM RES resulted in the downregulation of TNF-β/TNF-βR-induced epithelial–mesenchymal transition (EMT) in colorectal cancer cells, specifically HCT116, RKO, and SW480 cells. This effect was achieved through the specific suppression of the NF-κB pathway and focal adhesion kinase (FAK) [64]. The results suggest that RES may hold promise as a potential therapeutic agent for the treatment of colorectal cancer.

Furthermore, a ten-fold increase in RES (50 μM) was found to induce the degradation of tumor necrosis receptor-associated factor 6 (TRAF6). Overexpression of TRAF6 has been found to be closely associated with the epithelial–mesenchymal transition (EMT) process by activating the NF-κB pathway. In prostate cancer, overexpression of TRAF6 enhances EMT by upregulating the expression of the transcription factor SLUG. Therefore, it has been demonstrated that RES is capable of partially inhibiting the migration of prostate cancer cells (DU145 and PC3) [65]. Additionally, Rojo et al. have demonstrated that RES administered at doses ranging from 5 to 25 μM can enhance superoxide dismutase (SOD) activity, while concurrently suppressing NF-κB transcriptional activity and heparanase activity in gastric cancer cells (AGS and MKN45). This finding suggests that RES may have a role in reducing the invasive potential of gastric cancer cells [66]. Furthermore, Buhrmann et al. reported that RES can downregulate TNF-β-induced colorectal cancer cell (HCT116) proliferation by blocking TNF-β/TNF-β-receptor-induced NF-κB activation, resulting in activation of caspase-3 cleavage [67]. RES has been shown to have a dual effect in suppressing cancer proliferation and metastasis by inhibiting the NF-κB signaling pathway.

The STAT3 transcription factor has been the subject of extensive research due to its crucial function as a transcriptional regulator. STAT3 plays a critical role in several cellular processes, including oncogenesis, tumor growth and progression, and stemness [68]. In a study by Sun et al., RES was found to inhibit the proliferation and metastatic potential of cervical cancer cells (HeLa and SiHa) by deactivating STAT3 phosphorylation at Tyr705. The researchers observed that RES decreased the levels of N-cadherin, vimentin, MMP-3, and MMP-13 proteins while increasing the levels of E-cadherin protein in HeLa and SiHa cells [69]. These findings suggest the potential of RES as a therapeutic agent for cervical cancer. In a study by Ferraresi et al., it was demonstrated that RES can counteract lysophosphatidic acid (LPA)-induced malignancy in ovarian cancer cells (SKOV3, OVCAR3, OAW42, SKOV3-GFP-LC3) by inhibiting multiple signaling pathways, including PI3K-AKT, JAK-STAT, and Hedgehog pathways, as well as by restoring autophagy [70]. Likewise, Xu et al. demonstrated that RES counteracts hypoxia-induced gastric cancer invasion and EMT by inhibiting the Hedgehog pathway [71].

The Ras/RAF/MEK/ERK (MAPK) signaling pathway is a well-established pathway in cancer biology, and overactivation of this pathway is responsible for more than 40% of human cancer cases [72]. According to a study by Yang et al., RES has been reported to prevent interleukin 6 (IL-6)-induced gastric cancer metastasis by blocking Raf/MAPK signaling activation. In particular, the researchers observed that treatment with 20 μM RES significantly inhibited the invasion of gastric cancer cells (SGC7901) and suppressed the expression of MMP-2 and MMP-9, thereby interfering with the development of EMT. The efficacy of RES in inhibiting cancer cell metastasis was further demonstrated in mouse models. Specifically, HSC-39 cells were injected into each group of five mice via tail vein, and the mice were treated with 10 or 20 μM RES every 4 days for 3 weeks. The results showed that treated mice exhibited significant inhibition of cancer cell metastasis [73]. In addition, Xiao et al. demonstrated that RES can inhibit the malignant progression of CAL-27 oral squamous cell carcinoma cells by down-regulating the expression of vascular endothelial growth factor (VEGF), RGS5, CD105, and cell adhesion molecules ITGA5, ITGB1, and CD44 via the zinc finger protein 750/Ras-related C3 botulinum toxin substrate 1 (ZNF750/RAC1) signaling pathway, leading to a reduction in vascular normalization, metastasis, adhesion, and migration of CAL-27 cells [74]. Similarly, Chang et al. reported that RES can suppress the metastatic behavior of cisplatin-resistant human oral cancer cells by inhibiting the ERK/p-38 signaling pathway and MMP-2/9. The inhibition of migration and invasion abilities of cisplatin-resistant human oral squamous cell carcinoma cells were found to be dose-dependent with doses of 25 to 75 μM RES, while 50 μM RES treatment downregulated the expression of phosphorylated forms of ERK and p-38 in addition to MMP-2 and MMP-9 expression [75]. In renal cell carcinoma cells (ACHN and A498), Yang et al. showed that RES inhibits proliferation, migration, and invasion through Akt and ERK1/2 signaling pathways. RES was found to inhibit the expression of N-cadherin, vimentin, Snail, MMP-2, MMP-9, p-Akt, and p-ERK1/2, while increasing the expression of E-cadherin and TIMP-1 [76]. In a study by Daria et al., it was reported that RES is capable of binding and activating the Raf kinase inhibitor protein (RKIP) in colorectal cancer cells. RKIP functions as an inhibitor of the Raf-1, PI3K, and MAP kinase (MAPK) pathways. The combination of RES and RKIP targeting was found to help reduce metastasis in CRC cells (HT29 and HCT116) [77]. Targeting cancer cell metabolism through the application of AMP-activated protein kinase (AMPK) activators is considered to be one of the most plausible attempts [78]. In a study by Liu et al., it was reported that RES has been shown to inhibit proliferation and induce apoptosis in ovarian cancer cells (A2780 and SKOV3) by inhibiting glycolysis and targeting the AMPK/mTOR signaling pathway [79].

It has been noted that RES can inhibit the proliferation and migration of cancer cells by inhibiting TGF-β [80]. Zhang et al. reported that 50 μM of RES inhibited EMT in lung cancer cells (A549). TGF-β1-induced EMT leads to mitochondrial dysfunction, increased ROS production, and decreased mitochondrial membrane potential, ATP content, and mitochondrial complex protein expression. However, RES can protect mitochondria during EMT [81]. Remarkably, a study conducted on breast cancer corroborated the ability of RES to impede EMT by modulating the activity of transforming growth factor-beta 1 (TGF-β1). Additionally, RES was found to induce autophagy via the upregulation of SIRT3 and phosphorylated AMPK. The study revealed that the depletion of SIRT3 led to reduced levels of AMPK phosphorylation and autophagy-related proteins, thereby impairing the anticancer efficacy of RES. These findings suggest that RES-mediated inhibition of tumor progression is attributed to the involvement of the SIRT3/AMPK/autophagy signaling axis [82]. According to Deng et al., RES may inhibit EMT in gastric cancer cells by weakening the Hippo-YAP signaling pathway. The authors demonstrated that 5–20 μM of RES significantly inhibited the migration of gastric cancer cells (SGC-790), as well as the expression of marker proteins E-cadherin, vimentin, N-cadherin, and Snail that are associated with EMT occurrence [83].

The Wnt/β-catenin signaling pathway is a highly conserved evolutionary pathway known to regulate important cellular functions such as proliferation, differentiation, migration, genetic stability, apoptosis, and stem cell renewal. Yin et al. demonstrated that RES can inhibit the increased mobility of gastric cancer cells and reverse the progression of gastric cancer-derived MSCs-induced EMT by deactivating the Wnt/β-catenin signaling pathway [84]. Additionally, RES was found to decrease the expression of IL-6, IL-8, MCP-1, and VEGF genes, as well as protein secretion, and counteract the activation of the Wnt/β-catenin signaling pathway induced by gastric cancer-derived mesenchymal stem cells in gastric cancer cells with less β-catenin expression. Moreover, the expression of β-catenin, CD44, and CyclinD3 was reduced in transportation and cancer cells [85]. In addition, RES has been shown to exert antimetastatic effects by modulating microRNAs (miRNAs). miRNAs are a class of small non-coding RNAs that play a post-transcriptional regulatory role in gene expression by targeting messenger RNAs. Numerous studies have identified miRNAs as both promoters and inhibitors of metastasis [86]. Esposito et al. reported that RES inhibits the autophagy and metastasis of ovarian cancer cells (OVCAR3, OAW42, and KURAMOCHI) by downregulating miR-1305, which in turn regulates the downstream target ARH-1 [87]. In another ovarian cancer study, Yao et al. reported that RES induces apoptosis and inhibits proliferation and invasion in ovarian cancer cells through the miR-34a/Bcl-2 axis [88]. Furthermore, Su et al. showed that miR-155-5p promotes proliferation, invasion, and metastasis, and inhibits the apoptosis of gastric cancer cell lines (SGC7901, GES-1, MGC803 and AGS), while the addition of RES at a dose of 50 or 75 μM inhibited this effect [89]. Yang et al. reported that RES inhibited apoptosis, proliferation, invasion, and migration of human gastric cancer cells, which was induced through the MALAT1/miR-383-5p/DDIT4 signaling pathway [90]. Furthermore, Xiao et al. showed that RES induced the apoptosis of osteosarcoma cells (U2OS and MG63) by interfering with the miR-139-5p/NOTCH1 signaling pathway, thereby inhibiting the proliferation of cancer cells [91]. Lastly, RES also impacts the transportation of metal ions, consequently influencing cancer cell motility. According to Marco et al., RES was found to inhibit the adhesion/migration of MDA-MB-231 cells by suppressing Na^+^-dependent inorganic phosphate transporters [92]. Moreover, it was observed that TRPM7-mediated Mg^2+^ influx stimulates EMT and promotes cell migration in prostate cancer cells, while RES impairs TRPM7 function, thereby impeding prostate cancer metastasis [93].

**Table 1:** The molecular mechanism of resveratrol against cancer metastasis in preclinical research.

Cancer Type	Biological Test	IC_50_ or Dose	Molecular Mechanisms	References
Colorectal cancer	In vitro (HCT116)	5 µM	RES reduced the secretion of T-lymphocyte/fibroblast (TNF-β, TGF-β3) proteins, antagonized the T-lymphocyte/fibroblast-promoting NF-κB activation	[63]
Colon cancer	In vitro (HCT116, RKO, SW480)	5 μM	RES inhibiting NF-κB pathway and focal adhesion kinase (FAK) regulation	[64]
Prostate cancer	In vitro (DU145 and PC3)	50 μM	TRAF6/NF-kappa B/SLUG signaling pathway	[65]
Gastric cancer	In vitro (AGS and MKN45)	5–25 μM	RES increases SOD activity but decreases NF-κB transcriptional activity	[66]
Colorectal cancer	In vitro (HCT116)	5 μM	RES can block TNF-β/TNF-β-receptor-induced activation of NF-κB	[67]
Cervical cancer	In vitro (HeLa, SiHa); In vivo (BALB/C nude mice)	10–40 μM	RES suppressed inactivating phosphorylation of STAT3 at Tyr705	[69]
Ovarian cancer	In vitro (SKOV3, OVCAR3, OAW42, SKOV3-GFP-LC3)	100 µM	PI3K-AKT, JAK-STAT and Hedgehog pathway	[70]
Gastric cancer	In vitro (SGC-7901)	25, 50, or 100 μM	Hedgehog pathway	[71]
Gastric cancer	In vitro (SGC7901); In vivo (NOD/SCID mice)	50 or 100 μM; 10 or 20 μM (intratumorally injection)	Raf/MAPK signaling pathway	[73]
Oral cancer	In vitro (CAL-27)	10, 20, or 40 μM	ZNF750/RAC1 signaling pathway	[74]
Oral cancer	In vitro (CAR)	50 μM	ERK/p-38 signaling pathway	[75]
Renal carcinoma	In vitro (ACHN and A498)	132.9 ± 1.064 μM in ACHN, and 112.8 ± 1.191 μM in A498	Akt and ERK1/2 signaling pathways	[76]
Colorectal cancer	In vitro (HT-29 and HCT 116)	100 μM	RES binds and activates RKIP protein	[77]
Ovarian cancer	In vitro (A2780 and SKOV3); In vivo (BALB/c nude mice)	In A2780 and SKOV3 cells were 196.01 ± 33.09 μM and 56.99 ± 26.91 μM; 100 mg/kg/day, for 18 days (oral)	AMPK/mTOR signaling pathway	[79]
Lung cancer	In vitro (A549)	50 μM	RES can protect mitochondria during EMT occurrence	[81]
Breast cancer	In vitro (4T1); In vivo (BALB/c nude mice)	25 or 50 μM; 160 mg/kg (oral)	SIRT3/AMPK/autophagy signaling pathway	[82]
Gastric cancer	In vitro (SGC-790)	5–20 μM	weakening the Hippo-YAP signaling pathway	[83]
Gastric cancer	In vitro (AGC, HGC-27); In vivo (BALB/c nude mice)	(20-CM, i.p. for 35 days)	Wnt/β-catenin signaling pathway	[85]
Ovarian cancer	In vitro (OVCAR3, OAW42, KURAMOCHI)	10 µM	miR-1305 downregulation	[87]
Ovarian cancer	In vitro (OV-90 and SKOV-3)	100 µM	miR-34a downregulation	[88]
Gastric cancer	In vitro (SGC7901, GES-1, MGC803, and AGS)	50 or 75 µM	miR-155-5p downregulation	[89]
Gastric cancer	In vitro (SGC7901)	1 or 5 µM	MALAT1/miR-383-5p/DDIT4	[90]
Osteosarcoma	In vitro (U2OS and MG63)	5, 10 or 20 µM	miR-139-5p/NOTCH1	[91]
TNBC	In vitro (MDA-MB-231)	16.37 ± 4.72 μM	Na^+^-dependent Pi transporter is inhibited by RES	[92]
Prostate cancer	In vitro (DU145 and PC3)	123.90 ± 9.78 μM	Mg^2+^ influx via TRPM7 promotes cell migration by inducing EMT	[93]

### 6.2. Research Progress of Resveratrol as Anticancer Agent in Clinical Trials

Although the significant inhibitory effect of RES on cancer metastasis has been observed in the above preclinical studies, few clinical trials have been conducted to validate the efficacy of RES on the inhibition of tumor metastasis. Most currently available clinical studies in human subjects have examined the effective dosage and safety of RES in the treatment of cancer. Table 2 summarizes clinical trials of RES in healthy subjects or cancer patients. The clinical information on RES’s potential role in preventing cancer metastasis provides a valuable foundation for future research in this area. To ensure the safety of RES in clinical applications, a study was conducted by Espinosa et al. to investigate the effect of RES (1000 mg/day for 28 days) on circulating immune cells in healthy Japanese subjects. The results revealed a small yet significant increase in circulating γδ T cells and regulatory T cell numbers. Additionally, plasma levels of the proinflammatory cytokines TNF-α and MCP-1 were decreased, while plasma antioxidant activity was significantly increased compared to the corresponding baseline and control groups. However, some gastrointestinal adverse effects were reported in the treated group [94]. In another study, thirty participants were assigned to a low-RES diet and subsequently to one of three groups to consume 0.15–0.45 kg of grapes per day for 2 weeks. The short-term daily intake of grapes resulted in a reduction in Wnt signaling and mucosal proliferation, which may lower the risk of mutational events that can promote colon carcinogenesis [95]. In addition, a phase I clinical study was conducted to assess the safety of oral RES at proposed pharmacological doses. Ten healthy volunteers were assigned to each of four groups and administered a single dose of 0.5, 1, 2.5, or 5 g of RES. No serious adverse events were reported, and RES and its six metabolites were detected in plasma and urine. Peak plasma levels of RES were achieved 1.5 h post-dose, with the highest dose resulting in a level of 539 ± 384 ng/mL [96]. Zhou et al. conducted another clinical study to evaluate the effect of RES on drug-metabolizing enzymes. In this study, 42 healthy volunteers were given 1 g of RES daily for four weeks, which led to the suppression of phenotypic indicators of CYP3A4, CYP2D6, and CYP2C9, and the induction of CYP1A2. Some mild and transient side effects were observed in study participants [97]. Furthermore, a clinical trial involving healthy volunteers who received daily doses of RES at 0.5, 1.0, 2.5, or 5.0 g for 29 consecutive days demonstrated a significant reduction in plasma levels of IGF-1 and IGFBP-3, which play a key role in tumorigenesis. The most significant reductions were observed at a dose of 2.5 g, and gastrointestinal symptoms were mild to moderate at doses of 2.5 g and 5 g [98]. Overall, low doses of oral RES appear to be safe and well-tolerated in healthy individuals. In most clinical trials related to colorectal cancer, no side effects of RES were reported, and only one case reported mild gastrointestinal side effects of RES. A phase I randomized, double-blind pilot study demonstrated that micronization increased the absorption of RES in patients diagnosed with stage IV colorectal cancer and liver metastases. The results of this study have shown that micronized RES can increase the absorption of RES in patients diagnosed with stage IV colorectal cancer and liver metastases, thereby improving bioavailability. Therefore, the preparation can effectively overcome the shortcoming of low bioavailability of RES. Micronized RSE SRT501 5.0 g/d for 14 consecutive days resulted in detectable RES (up to 2287 ng/g) in the liver tissue of subjects. The apoptosis marker cleaved caspase-3 was significantly increased by 39% in SRT501-treated malignant liver tissues compared to placebo-treated patients. Unfortunately, some patients experienced gastrointestinal side effects [99]. Cai et al. conducted a study to investigate the distribution of radiolabeled RES in the malignant and non-malignant tissues of colorectal cancer patients. The patients were administered 1 g/day of ^14^C-labeled RES for six days, and the total RES in malignant tumor tissues ranged from 3.0 to 376.0 nmol/g. RES was also detected in non-malignant colonic mucosa and muscle tissue. Furthermore, a low dose of 5 mg/day was found to elevate biomarkers of oxidative stress and activate maximal autophagy and AMP-activated protein kinase (AMPK) in colorectal tumor tissue. These findings suggest that RES may have potential as a therapeutic agent for colorectal cancer [100]. Additionally, a phase I clinical trial was conducted to investigate the effect of RES and grape powder on the expression of Wnt pathway target genes in the colonic mucosa and colon cancer. Eleven patients received different doses of RES (20 mg/80 mg/160 mg) orally before surgical resection of the tumor. At 14 days, it was found that RES did not inhibit the Wnt signaling pathway in colon cancer. However, a combination of 20 mg RES with grape powder was found to inhibit the nor-colonic mucosal Wnt signaling pathway [101]. The proliferative effect and safe dose test of colon cancer treatment involved administering RES 0.5 or 1.0 g to 20 patients with histologically confirmed colorectal cancer before operation, eight times a day. Normal and malignant biopsy samples were obtained prior to dosing. Quantification was performed using the HPLC/UV method. RES and resveratrol-3-o-glucuronide were recovered from tissues at maximal mean concentrations of 674 nmol/g and 86.0 nmol/g, respectively. The levels of RES and its metabolites were consistently higher in tissues derived from right hemicolectomy than in tissues derived from left hemicolectomy. RES reduced tumor cell proliferation by 5%. Oral administration of 0.5 or 1.0 g RES per day produced levels sufficient to induce anticancer effects in the human gastrointestinal tract [102]. In summary, RES has been shown to be well-tolerated in various preclinical and clinical studies. However, further research is needed to determine the optimal and safe dosage for humans, as well as its potential long-term effects. A double-blind, randomized, placebo-controlled parallel trial was conducted on 22 men with biochemically recurrent prostate cancer. The study evaluated the effect of a mixture of high-dose RES (30 mg) on prostate-specific antigen doubling time (PSADT). Although a non-significant prolongation of PSADT was reported, the low doses used in the study were likely the cause of the lack of effect. The investigators acknowledged that higher doses may be necessary to achieve the desired outcome [103]. In a separate Phase I and II clinical program, Dr. Paller and his team evaluated the use of low-microgram doses of MPX capsules, a ground muscadine grape (Vitis rotundifolia) skin containing ellagic acid, quercetin, and RES, as a treatment option for patients with biochemically recurrent prostate cancer. The initial low (0.5 g) and high (4 g) doses of MPX were determined to be safe. However, chronic administration of subthreshold microgram doses was associated with side effects, and during 12 months of treatment, there was no change in PSADT indicating disease progression [104,105]. Based on the results of the study, it seems that cancer patients who take a combination of multiple natural products in capsule form experience fewer side effects compared to those who take RES alone orally. While RES may be present in small quantities in these capsules, the multicomponent therapy approach allows for multiple targets to be addressed simultaneously, while also minimizing the risk of toxic side effects that may result from high doses of a single drug. This approach helps to ensure medication safety. Clinical trials of RES for the treatment of breast cancer have demonstrated promising results. In a double-blind, randomized trial, 39 women at high risk of breast cancer received oral *trans*-RES capsules at a dose of 5 or 50 mg twice daily for 12 weeks. The treatment was well-tolerated with few side effects, and trans-RES levels in serum samples increased significantly in the high-dose group compared to the placebo group after 4 and 12 weeks, indicating a dose-dependency [106]. Additionally, dietary plant extract capsules containing approximately 161.6 mg RES per day resulted in nanomolar concentrations of plasma RES. RES and its metabolites were found to be more concentrated in malignant tumors than in normal tissues, potentially due to their lipophilicity [107]. RES has also been studied for the prevention of breast cancer, with a 1 g/day intervention over 12 weeks in 40 subjects, of which 6 withdrew due to adverse events, and 34 completed the intervention [108]. However, administration of a dose of 5 g/day in myeloma patients resulted in unexpected nephrotoxicity, indicating the need for further research to determine the optimal RES dose for the treatment of breast cancer [109].

## 7. Pharmacokinetics and Toxicity Studies of Resveratrol

### 7.1. Issues of Pharmacokinetics

The low bioavailability and high-dose potential nephrotoxicity associated with RES are major bottlenecks for its further development as an anticancer drug. Oral RES has been reported to be less than 5% due to extremely rapid phase II metabolism such as gut/hepatic glucuronidation and sulfate conjugation [11,110].

Although the *trans*-isomer is more biologically active than the *cis*-isomer, its use is very limited due to the extensive metabolism of *trans*-RES. *Trans*-RES and its related conjugates are mainly distributed in the liver and kidney, the primary site of the tumor, and ^14^C-labeled radioactive RES was also observed in the stomach and intestinal tract after oral administration, suggesting that these tissues are “resveratrol pools” [111]. Furthermore, Dr. Walle demonstrated that the rapid and extensive intestinal or hepatic biotransformation of RES results in extremely limited bioavailability. The pharmacokinetics of ^14^C-RES were investigated in a study that utilized both oral and intravenous administration. Oral administration of ^14^C-labeled RES 25 mg was given to six healthy volunteers, resulting in a minimum absorption rate of 70%, a total urine radioactive recovery of 53.4–84.9%, and a t_1/2_ of 9.2 h. Intravenous injection of 0.2 mg ^14^C-labeled RES was given to five healthy volunteers, resulting in a total radioactivity recovery in total urine of 42.3–83.2%, and a t_1/2_ of 11.4 h. These findings suggest that there is a significant first-pass effect for orally administered RES [11].

In a double-blind, randomized, placebo-controlled study involving ten healthy adults, RES was administered orally to eight patients at varying doses of 25, 50, 100, or 150 mg, six times daily for a total of 13 doses. Two patients received placebo capsules. Following administration, peak plasma concentration (C_max_) of *trans*-RES were observed at 3.89, 7.39, 23.1, and 63.8 ng/mL, respectively, with average areas under the plasma concentration–time curves of 3.1, 11.2, 33.0, and 78.9 ng·h/L. The half-life of *trans*-RES was found to be 1–3 h after a single dose and 2–5 h after repeated doses. Trough concentration (C_min_) of 1 ng/mL and 3 ng/mL were observed after 25 mg and 50 mg doses, respectively, while 100 mg and 150 mg doses resulted in C_min_ values of 3 ng/mL and 10 ng/mL, respectively. At 8:00 a.m. and 12:00 p.m., the C_min_ of subjects receiving oral 150 mg *trans*-RES was 9.24 ng/mL and 5.84 ng/mL, respectively. Repeated doses of *trans*-RES were well-tolerated and showed high absorption of the morning dose [112]. Tests of 1 g of *trans*-RES in two-week human urine samples showed that RES was rapidly metabolized by sulfate and glucuronide conjugation and excreted in urine [113]. Similarly, an analysis of patients receiving 5 mg or 1 g of RES orally (2 weeks before prostate biopsy) found low levels of RES but high levels of glucuronic acid and sulfate metabolites [114]. Based on the above results, the low bioavailability of RES is due to its intense phase II metabolism in the gut and liver rather than its low water solubility. Nanotechnology has emerged as a promising approach to address the physicochemical and pharmacokinetic limitations of RES. Various nanoformulations, such as liposomes, solid lipid nanoparticles, polymeric nanoparticles, and cyclodextrins, have been developed to specifically target cells and reduce toxicity by lowering the required doses [115]. Despite the potential benefits of RES nanoencapsulation, it remains an emerging field with several challenges that need to be addressed, such as the long-term safety of nanoparticles, their interaction with biological systems, and the need to develop stable and reproducible nanoformulations with high RES loading capacity. To enhance the effectiveness of hydrophobic drugs such as resveratrol, alternative nanoparticles need to be explored, and research on nanomaterial synthesis and inorganic nanoparticles need to be increased. Notably, recent studies have reported that RES nanoformulations can inhibit cancer metastasis, indicating their therapeutic potential. For instance, in B16F10 melanoma-bearing C57BL/6J mice, RES-loaded oil core Poly(ε-caprolactone) (PCL) nanocapsules showed promising results by increasing necrosis and inflammation in tumor tissue, reducing pulmonary hemorrhage, and preventing lung metastasis [116]. Similarly, Pradhan et al. reported that nanoformulated resveratrol inhibited metastasis and angiogenesis by reducing inflammatory cytokines in oral cancer cells [117]. These findings suggest that RES nanoencapsulation could be a promising alternative therapy for cancer metastasis.

### 7.2. Toxicity Effects of Resveratrol

The incidence and severity of toxic effects associated with RES administration are largely dependent on the dosage administered. Reports on the toxic effects of RES have focused on nephrotoxicity and gastrointestinal side effects. The dosage of RES administered is a crucial factor in determining the occurrence of toxicity, and there are typically few or no drug-related adverse effects when RES is administered in a single dose of less than 1 g [118,119]. In healthy subjects, mild and transient adverse events such as diarrhea, nausea, vomiting, abdominal distension, abdominal cramping, headache, and rash were observed when 0.5 g/day of RES was taken for one month. Another study involving 40 healthy volunteers who ingested 0.5, 1.0, 2.5, or 5.0 g of RES per day for 29 days demonstrated that serious gastrointestinal side effects only occurred at doses higher than 2.5 g [98]. In a phase II clinical trial of eight healthy subjects taking trans-RES 2 g twice daily, six of eight subjects developed diarrhea [120]. In fact, an open-label trial showed that high doses of RES caused severe gastrointestinal effects, with four subjects developing intolerance after receiving high doses of RES at 5 g/d. Diarrhea occurred in 71% of subjects given the 2.5 g/d RES dose [121]. A phase II trial in subjects with multiple myeloma showed severe nephrotoxicity in five subjects receiving RES at a dose of 5 g/day [109]. Among them, abdominal discomfort was the main toxicity reported. It should be noted that the longest duration of RES administration reported in the literature is 1 month, indicating the need for further investigations into the potential toxicity and safety of RES when used for longer treatment periods.

## 8. Conclusions

Natural products, including plant extracts and herbal remedies, have long been used in traditional medicine systems worldwide [122]. In recent years, there has been a growing interest in the use of natural products as potential treatments for a wide range of diseases, including cardiovascular disease, cancer, and immune dysfunction. This interest is driven by the perception that natural products are safe and effective, and by the increasing awareness of the limitations and side effects of conventional pharmaceuticals. Natural products offer a diverse and complex array of chemical compounds that can target multiple biological pathways, making them attractive candidates for drug development. The potential of natural products, such as RES, in treating diseases such as cancer, cardiovascular disease, and immune dysfunction is gaining popularity. RES has shown efficacy in inhibiting cancer metastasis, the leading cause of cancer death. However, before it can be developed into a form of treatment for patients with advanced cancer and metastatic symptoms, several issues need to be addressed. One of the main issues is the low bioavailability of RES, which can be addressed by developing more attractive nanodelivery systems, such as mucoadhesive nanocarriers that can deliver the drug directly to the circulation system through the nasal passageway. In addition, origins-on-chips technology can provide new strategies for drug delivery route screening and drug metabolism assessment in multiple organs. Designing accurate and multifaceted animal models of cancer metastasis is also necessary to investigate the interaction between different types of cells, and clinical trials are needed to provide reasonable predictions on the efficacy of RES in patients with several cancers. To overcome the bottlenecks of RES in further development in cancer therapy, more research is needed on the discovery of new derivatives and novel formulations of RES. Strategies to avoid low bioavailability include developing more potent analogues such as Pterostilbene and using new dosage forms such as nanotechnology. In summary, RES is expected to be a promising natural product for the prevention and treatment of metastatic cancer, but further research is needed to develop more effective analogues or formulations and suitable models for studying metastases in various cancer types.

## Figures and Tables

**Table 2:** Chemoprevention of resveratrol in a clinical trial.

Phase	Tumor Type	Subjects	Administration	Dose/ Duration	Results	References
I	-	9 (healthy)	Oral	1 g, 28 days	Gastrointestinal side effects	[94]
I	-	30 (healthy)	Oral (grapes)	0.15/0.3/0.45 kg (~7.5/15/22.5 mg RES), 2 weeks	No side effects	[95]
I	-	40 (healthy)	Oral	0.5/1/2.5/5 g	Peak plasma levels of RES at the highest dose were 539 ± 384 ng/mL	[96]
I	-	42 (healthy)	Oral	1 g, 4 weeks	RES intervention inhibited the phenotypic indices of CYP3A4, CYP2D6, and CYP2C9 and induced the phenotypic indices of 1A2	[97]
Ⅱ	-	40 (healthy)	Oral	0.5/1/2.5/5 g, 29 days	Only 2.5 and 5 g doses causing mild-to-moderate gastrointestinal symptoms	[98]
I	Colorectal cancer (hepatic metastasis)	9 (patients)	Oral (SRT501)	5 g, 14 days	Gastrointestinal side effects, cleaved caspase-3 was significantly increased by 39%	[99]
I	Colorectal cancer	24 (patients)	Oral	5 mg/1 g, 6 days	RSE ranged from 3.0 to 376.0 nmol/g in malignant tumor tissue	[100]
I	Colon cancer	8 (patients)	Oral (Tablet/Grape Powder)	20/80/160 mg (RES), 2 weeks	Not significantly inhibit the Wnt pathway in malignant colonic tissue	[101]
I	Colorectal cancer	20 (patients)	Oral	0.5/1 g, 8 days	0.5 or 1.0 g of RES per day no side effects	[102]
I	Prostate cancer	22 (patients)	Oral	~30 mg, 12 weeks	RES (~30 mg) reported a non-significant prolongation of PSADT	[103]
I/Ⅱ	Prostate cancer	14 (patients)	Oral (4.4 μg RES for per capsule)	1/2/3/4 g (8.8/17.6/26.4/35.2 μg RES), 28 days	4 patients developed gastrointestinal side effects in the high-dose group	[104]
Ⅱ	Prostate cancer	125 (patients)	Oral (4.4 μg RES for per capsule)	0.5/4 g (4.4/35.2 μg RES), 12 months	One patient developed gastrointestinal side effects in the high-dose group	[105]
I	Breast cancer	39 (menopausal women)	Oral	5/50 mg, 12 weeks	No side effects in the subjects, and trans-RES was detected in 20% of the subjects’ serum samples	[106]
I	Breast cancer	19 (patients)	Oral	161.55 mg, 6 ± 2 days	RES and its metabolites are more concentrated in malignant tumors compared to normal tissues	[107]
I	Breast cancer (revelation)	40 (healthy)	Oral	1 g, 12 weeks	6 patients developed side effects leading to withdrawal	[108]
Ⅱ	Multiple myeloma	24(patients)	Oral (SRT501)	5 g, 21 days	Nephrotoxicity leads to study termination	[109]
